# Surgical effect of the medial wall resection of the cavernous sinus for functional pituitary adenomas

**DOI:** 10.3389/fsurg.2024.1439909

**Published:** 2024-12-06

**Authors:** Xiao Liang, Zhuoqun Li, Mengyang Xing, Wenbo Gao, Pengfei Liu

**Affiliations:** Department of Neurosurgery, Binzhou Medical University Hospital, Binzhou, Shandong, China

**Keywords:** cavernous sins, function pituitary adenoma, aggressive pituitary adenoma, outcome, medial wall removal

## Abstract

**Background:**

The surgical treatment of pituitary adenomas (PAs) is aimed at achieving maximal tumor resection, relieving the compression, and correcting the disorders of pituitary hormones. Parasellar dural invasion is a primary factor in the failure of the surgery. By comparing the two operations of tumor excision combined with resection of the medial wall of the cavernous sinus (MW) and simple tumor excision, we further confirmed the clinical effectiveness and safety of the resection technique of the MW.

**Methods:**

41 patients with functional pituitary adenoma (FPA) were divided into two groups according to the operation. The experimental group consisted of 20 patients who underwent tumor excision combined with resection of the MW via endonasal transsphenoidal approach and 21 patients who underwent simple pituitary tumor excision as the control group. Both groups were followed up for 12 months and matched for age, sex, BMI, tumor type, Knosp grade, maximum tumor diameter, hypertension, diabetes, and coronary disease. Perioperative-related indicators, biochemical remission rates, tumor recurrence rates, and complications were assessed.

**Results:**

A total of 21 medial walls were removed in 20 patients, 15 (71%) specimens had pathologically confirmed tumor invasion. Biochemical remission rates and average operative duration in the experimental group were more than in the control group (*P* < 0.05). The remaining perioperative indicators, complications, and tumor recurrence rates had no statistically significant difference (*P* > 0.05).

**Conclusion:**

The technique of the MW removal via endonasal transsphenoidal approach for FPAs is safe and effective, with a high biochemical remission. The average operative duration for MW removal may be longer than that for simple tumor excision.

## 1 Introduction

Pituitary adenomas (PAs) are common benign intracranial tumors, accounting for about 10%–15% of all intracranial tumors (1, 2), classified into functional pituitary adenomas (FPAs) and non-functional pituitary adenomas (NFPAs) depending on whether hormones are hypersecretion, with FPA accounting for approximately 65%–85% (3). Their symptoms, such as headache, visual field impairment caused by tumor space-occupying effects, acromegaly, amenorrhea, centripetal obesity caused by abnormal hormone secretion, and others, seriously affect patients’ quality of life (4). Surgical operation is the primary treatment for FPAs. Pituitary tumor excision via endoscopic endonasal transsphenoidal approach can reduce operative trauma. It provides better illumination and a wider field of view, making identifying tumor and normal tissue under the endoscope easier and ensuring complete tumor resection. It is currently the primary operative method for PAs in clinical practice (5). Postoperative hormone level is the leading indicator reflecting the operative effectiveness of FPAs. The objective of surgical treatment of FPAs is achieving maximal tumor resection, relieving the compression, correcting the disorders of pituitary hormones, and reducing postoperative recurrence under the premise of ensuring safety (6). However, even if all visible tumors are resected during operation, a certain proportion of FPA patients still experience recurrence after surgery and low biochemical remission rates. The reason for this phenomenon may be that the tumor has invaded the medial wall of cavernous sinus (MW) (7, 8). The systematic explanation of MW-related anatomy has provided a basis for MW removal in recent years (9). This study, comparing the biochemical remission rate, tumor recurrence rate, and other related indicators of the two surgical methods by retrospectively analyzing, aims to confirm the safety and effectiveness of MW resection further.

## 2 Materials and methods

### 2.1 Patient population

The Affiliated Hospital of Binzhou Medical College database of clinical was reviewed to identify 20 patients with FPAs who underwent MW removal in addition to tumor excision via endoscopic endonasal transsphenoidal approach as the experimental group. Inclusion criteria included (1): without a history of previous pituitary tumor excision; (2) a diagnosis of FPA based on laboratory evidence of hormone hypersecretion and histopathologic analysis; (3) destruction or perforation of MW observed during operation, the adhesion of tumor elements to the MW or PA without intact pseudocapsule, and in direct contact with the MW. Exclusion criteria included: (1) a history of prior surgery; (2) a diagnosis of non-functional pituitary adenoma; (3) with other sellar region disease; (4) surgery not via endoscopic endonasal transsphenoidal approach or existing operative contraindications.

21 Patients with FPAs from the same database matched for age, sex, BMI, tumor type, Knosp grade, maximum tumor diameter, hypertension, diabetes, and coronary disease were identified as the control group, who underwent just tumor excision without MW removal. All patients were followed up for 12 months. No statistical difference was noted in these variables between the two groups (Table 1).

**Table 1:** Patient and tumor characteristics.

Characteristic	Control group		Experimental group		*P* value
	No. of cases	%	No. of cases	%	
Age (y)	46.95 ± 10.72		45.00 ± 12.81		0.599^a^
Sex					
Male	8	38	11	55	0.278^b^
Female	13	62	9	45	
BMI	25.78 (22.99–29.52)		25.49 (22.43–27.99)		0.876^c^
Knosp grade					
0	1	5	1	5	1.000^d^
1	4	19	3	15	
2	3	14	4	20	
3	7	33	7	35	
4	6	29	5	25	
Tumor type					
GH	10	48	12	60	0.806^d^
PRL	9	43	6	30	
ACTH	2	10	2	10	
Largest diameter of tumor (cm)	2.79 ± 0.91		2.48 ± 0.90		0.277^a^
Hypertension, yes	6	29	2	10	0.269^b^
Diabetes, yes	7	33	8	40	0.658^b^
Coronary disease, yes	2	10	1	5	1.000^d^

^a^ Independent sample *T*-test.

^b^ Chi-square test.

^c^ Mann-Whitney *U*-test.

^d^ Fisher's exact test.

### 2.2 Surgical technique

The traditional endoscopic endonasal transsphenoidal approach has been widely described and will not be further discussed here. The anesthesia was administered using a combined intravenous and inhalation anesthesia method. After sufficient anesthesia, the patient was placed in a supine position with the upper body raised about 15° (to reduce intraoperative bleeding), and the surgeon stood on the patient's right side. After routine disinfection of the nasal mucosa and surrounding skin, epinephrine-soaked cotton pads were placed on the surface of the nasal mucosa to constrict and reduce intraoperative mucosa bleeding.

**Figure 1:**
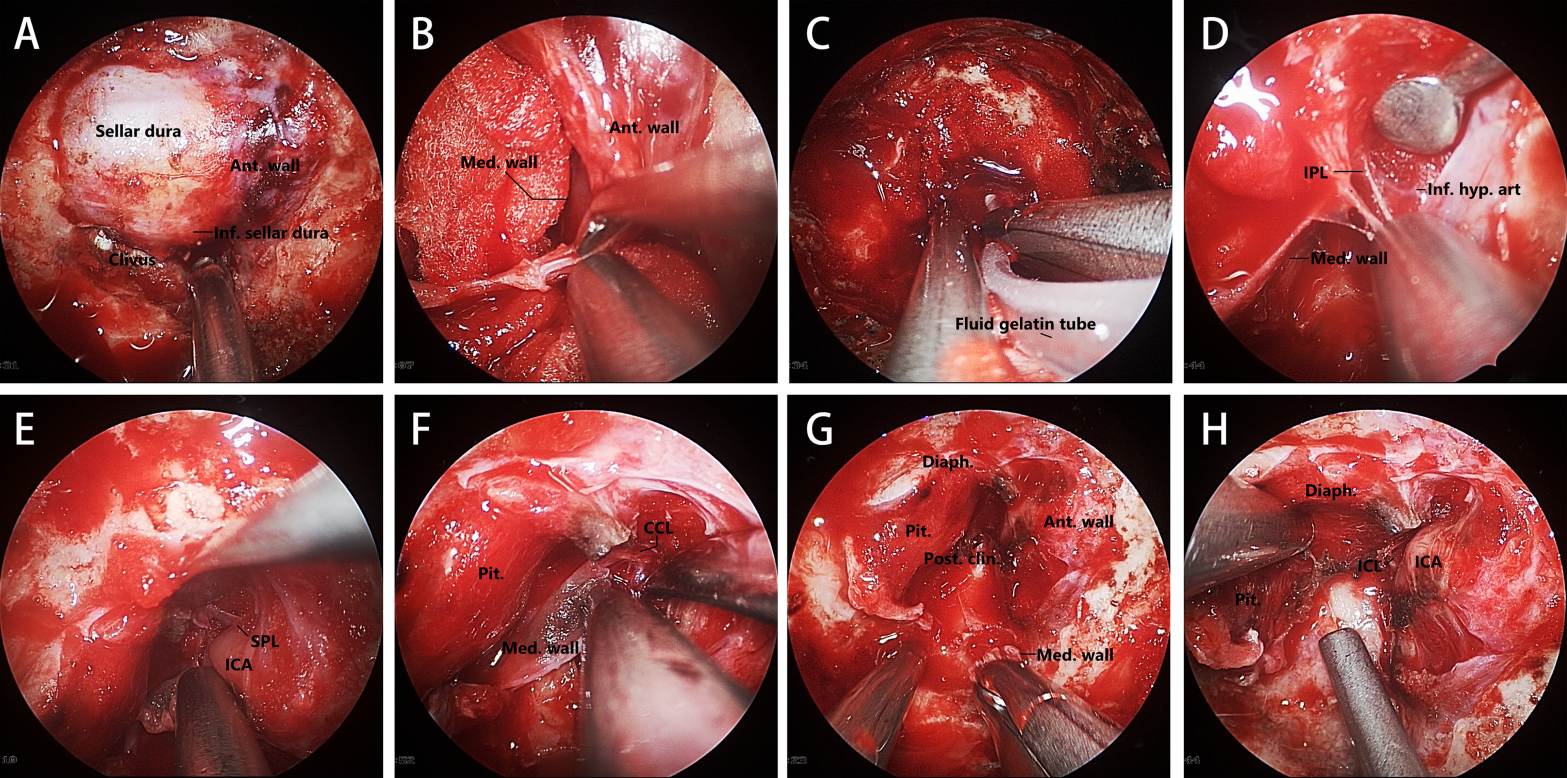
Key steps in this MW resection technique. **(A)** Sellar and parasellar dural exposure. **(B)** Opening the anterior wall of the cavernous sinus by separating the meningeal layer from the periosteal layer. **(C)** Fluid gelatin injected into the gap between the two dural layers. **(D–F)** Transcavernous Approach to identifying and dissecting the structure anchoring the MW. **(G, H)** Exposure of the ICL and the MW removing completely. Ant. Wall, anterior wall; Inf., inferior; Med. wall, medial wall; Inf. hyp. art, inferior hypophyseal artery; Pit., pituitary gland; Diaph., diaphragma sellae; Post. clin., posterior clinoid.

Exposure of the sellar dura should be enlarged upward to the distal dural ring and the tuberculum sellae, downward to reveal the paraclival segment of internal carotid artery (ICA), and on both sides to include the anterior wall of the cavernous sinus on the lesion side (Figure 1A).

The resection of the MW of the cavernous sinus requires careful identification of surrounding structures. We used a high-resolution endoscope (N780, Qingdao NovelBeam Technology Company) to assist us in completing this process. The first step of MW resection of the cavernous sinus is to open the anterior wall of the cavernous sinus, which can fully expose the cavernous segment of the ICA, facilitating the searching and identifying of the structures related to anchoring the MW, and timely dealing with intraoperative bleeding. The position to enter the cavernous sinus is usually at the lowest point of the anterior wall of the cavernous sinus (Figure 1B). Before opening the anterior wall, Doppler ultrasound should be used to confirm the route of the ICA to avoid damage. The incision of the anterior wall should be expanded to the contralateral side of the tumor to expose the MW fully, and fluid gelatin should be injected into the sinus to prevent venous bleeding (Figure 1C). The inferior parasellar ligament (IPL) is usually the first ligament encountered, and its transection will facilitate the movement of the medial wall. The inferior hypophyseal artery (IHA), a branch of the ICA, is often located behind the IPL (Figure 1D). It should be identified and electrocoagulated intraoperatively to prevent tearing of the ICA during movement of the MW. The next ligament to be identified and transected is the superior parasellar ligament (SPL), which connects the medial wall to the horizontal portion of the cavernous ICA (Figure 1E). After that, Separating the dura covering the dorsum sellae upwards until encountering the caroticoclinoid ligament (CCL). Then, the MW needs to be separated from the inner layer of diaphragma sellae (sellar roof), which may damage the diaphragma sellae and cause cerebrospinal fluid (CSF) leakage. Then, the MW is only connected to the ICA by the CCL and the dural attachments that make up the proximal dural ring (Figure 1F). As all CCL fiber bundles are sharply separated in an anterior-to-posterior direction, the MW can be safely separated from the cavernous sinus. When the CCL is completely disconnected, the posterior interclinoid ligament (ICL) and the dura of oculomotor triangle should be visible, which can be regarded as a sign of complete CCL disconnection (Figures 1G,H).

### 2.3 Data collection and definition

#### 2.3.1 Surgical factors or intraoperative complications

The operation time, postoperative hospitalization time, intraoperative CSF leakage rate, blood loss, and tumor resection rate were compared between the two groups of patients. Among them, blood loss (ml) = the amount of fluid in the drainage tube—the total amount of intraoperative irrigation fluid, result rounding off. Postoperative hospitalization time refers to the time from the end of the patient's operation to discharge, with data counted as one day for more than half a day and 0.5 days for less than half a day. Intraoperative CSF leakage refers to the clear fluid outflowing from the diaphragma sellae during the operation. Intraoperative endoscopic observations and postoperative MRI examinations were used to determine tumor residual.

#### 2.3.2 Post-operative complications

These include postoperative CSF leakage, intracranial infection, postoperative nasal hemorrhage, and diabetes insipidus. Postoperative CSF leakage refers to the outflow of clear liquid from the nasal cavity of the patient after surgery, and the gauze infiltration test shows stratification. The diagnosis of intracranial infection is based on postoperative CSF laboratory examination. Nasal hemorrhage refers to hemorrhage that requires surgical intervention. Diabetes insipidus refers to urine volume >4,000 ml/24 h after surgery, with urine specific gravity ≤1.005.

#### 2.3.3 Biochemical remission

We used the criteria for biochemical remission of functioning tumors as set forth by the *Consensus of Chinese Experts on Surgical Treatment of Pituitary Adenoma* (10). For acromegaly, the criterion was the random GH values <1 ug/L (If GH is ≥1.0 ug/L, an OGTT-GH suppression test needs to be performed), and the IGF-1 values decreased to match the gender and age. For prolactinomas, remission was defined as the PRL values <15 ug/L for males and <20 ug/L for females without the treatment of dopamine receptor agonist. For Cushing's disease, it was the serum cortisol <20 ug/L within 2d after the operation. Within half a year after the operation, the serum cortisol, 24-hour urine-free cortisol, and ACTH are at normal levels, and the clinical symptoms are relieved. FPA that meets the above criteria for 6 months is a biochemical remission.

#### 2.3.4 Histopathologic analysis

All resected tumor tissues were pathologically confirmed as functioning tumors after surgery. The presence or absence of MW invasion was diagnosed by a neuropathologist.

#### 2.3.5 Recurrence

No recurrence was defined as no evidence of tumor residue on dynamic enhanced MRI during the last follow-up, endocrine work-up consistent with biochemical remission criteria, and no corresponding clinical manifestations of hormone hypersecretion. On the contrary, it was defined as a recurrence.

### 2.4 Data analysis

Statistical analysis was performed using SPSS 26.0 software. Count data were analyzed using the chi-square test or Fisher's exact test and expressed as [cases (%)]. Measurement data were analyzed using an independent samples *T*-test expressed as (mean ± standard deviation), or Mann-Whitney *U*-test expressed as the median (25% quantile—75% quantile), depending on the normality of the distribution as determined by a Shapiro-Wilk test and Kolmogorov-Smirnov test. A *P*-value of <0.05 was considered statistically significant.

## 3 Result

### 3.1 Surgical factors and intraoperative complications

The incidence of intraoperative CSF leakage, intraoperative blood loss, postoperative hospitalization time, and tumor total resection rate had no statistically significant differences between the two groups of patients (*P* > 0.05). The average operation time of the experimental group was longer than that of the control group, and the difference was statistically significant (*P* < 0.05 Table 2).

**Table 2:** Surgical factors and intraoperative complications in the two groups.

Characteristic	Control group		Experimental group		*P* value
	No. of cases	%	No. of cases	%	
Operation time (h)	4.50 (3.42–6.57)		5.65 (4.29–8.02)		**0.046** ^a^
Postoperative hospitalization time (d)	11.5 (8.5–13.25)		10.5 (8–18.75)		0.657^a^
Blood loss (ml)	100 (50–250)		100 (100–200)		0.243^a^
Intraoperative CSF leakage (Y/N)	9/12	43/57	14/6	70/30	0.080^b^
Gross total resection (Y/N)	17/4	81/19	18/2	90/10	0.706^b^

^a^ Mann-Whitney *U*-test.

^b^ Chi-square test.

Bold value indicates that the *P*-value is less than 0.05.

### 3.2 Post-operative complications and outcomes

The incidence of postoperative CSF leakage, postoperative nasal hemorrhage, intracranial infection, or diabetes insipidus between the two groups showed no statistically significant (*P* > 0.05). The biochemical remission rate of the experimental group (75%) was significantly higher than that of the control group (38%), and the difference was statistically significant (*P* < 0.05). with no statistically significant difference in the recurrence rate between the two groups (*P* > 0.05 Table 3).

**Table 3:** Post-operative complications and outcomes in the two groups.

Characteristic	Control group		Experimental group		*P* value
	No. of cases	%	No. of cases	%	
Postoperative CSF leakage (Y/N)	2/19	10/90	2/18	10/90	1.000^a^
Nasal hemorrhage (Y/N)	0/21	0/100	2/18	10/90	0.232^b^
Intracranial infection (Y/N)	3/18	14/86	6/14	30/70	0.402^a^
Diabetes insipidus (Y/N)	8/13	38/62	4/16	20/80	0.203^a^
Biochemical remission (Y/N)	8/13	38/62	15/5	75/25	**0** **.** **017** ^a^
Recurrence (Y/N)	4/17	19/81	2/18	10/90	0.706^a^

^a^ Chi-square test.

^b^ Fisher's exact test.

Bold value indicates that the *P*-value is less than 0.05.

**Figure 2:**
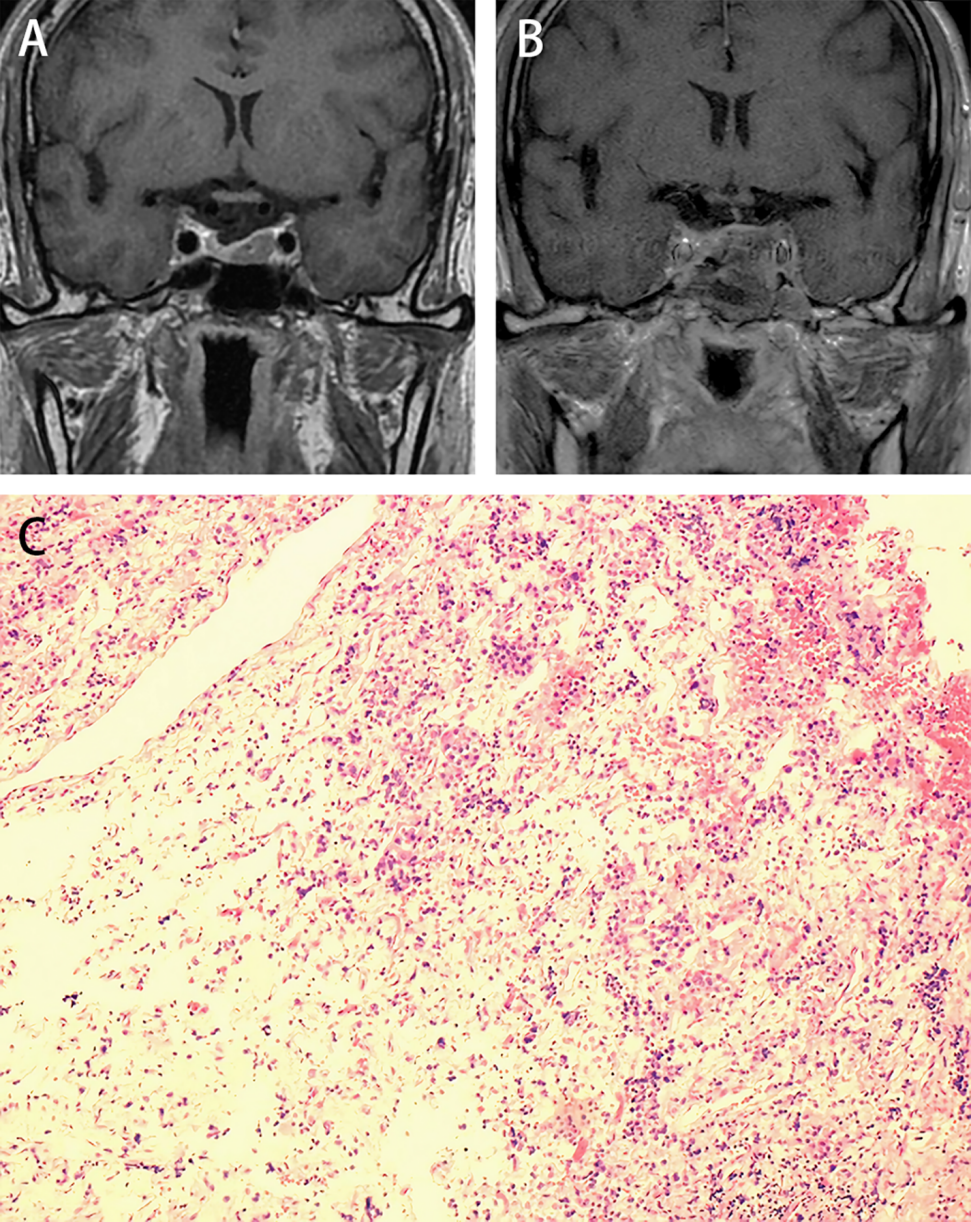
Illustrative case: A female, 64 years old, diagnosed with acromegaly, presented with a widening jaw, enlarged hands, and tachycardia et al. **(A)** Preoperative MR dynamic enhancement examination shows a left-sided adenoma, with Knosp Grade I. **(B)** Post-operative cranial MRI at the last follow-up shows no evidence of tumor recurrence. **(C)** The MW specimen stained with H&E (10 × 10) shows basophilic neuroendocrine cells scattered in the fibrous connective tissue. The random GH values dropped from 9.59 ug/L to 0.59 ug/L on the first day after surgery.

A total of 21 medial walls were removed in 20 patients, of which 15 patients (75%) were histopathologically confirmed with tumor invasion after surgery (Figure 2).

## 4 Discussion

### 4.1 Efficacy and safety of MW resection technique

Previous studies suggested that the invasion of the tumor to the parasellar structures is the primary factor that affects the surgical effect of PAs (7, 8). In our study, 15 patients out of 20 in the experimental group achieved complete biochemical remission after surgery, representing a significant improvement compared to 8 patients out of 21 in the control group (*P* < 0.05), indicating that MW resection has a definite positive effect on treating FPAs, indirectly reflecting the occult invasion of tumor cells to the MW. In the studies by Ishida et al. (11), Miranda et al. (12), and Nagata et al. (13), the biochemical remission rates of patients using this technique were 94% (96/102), 97% (34/35), and 93% (13/14) respectively, which were higher than the 75% (15/20) in our study. Omar et al. (14) obtained similar results to ours, with a remission rate of 75% (12/16). This result is because the groups in Miranda and Nagata's studies did not include patients with Knosp grade 4. Moreover, some patients without definite MW invasion were also included in Ishida's study. This point was reflected in the MW's pathological reports.

We further analyzed 11 patients with knosp grade 4 in our study. Among the 6 patients in the control group, only 1 achieved biochemical remission, with 2 cases of recurrence, corresponding to 2 cases of remission and 1 case of recurrence in the experimental group. The results indicated no statistical difference in biochemical remission and recurrence between the two groups. This suggests that for patients with Knosp grade 4, the efficacy of MW resection may be limited. The primary reasons are the extensive invasion of the cavernous sinus, which makes complete tumor removal difficult, a lower rate of gross total resection, and some “occult” invasion may not have been identified. Based on this, the MW resection for patients with knosp grade 4 should be carefully assessed.

In our study, the average operation time of the experimental group was longer than that of the control group, which was predictable because the MW resection technique requires multiple applications of Doppler ultrasound to locate the ICA, strict hemostasis, careful identification and separation of the relevant tissue structures that anchor the MW during operation, all of which undoubtedly increase the operation time.

Compared with traditional surgical methods, the MW resection of the cavernous sinus expands the resection range further. It increases the surgical manipulation of the diaphragm sellae, theoretically increasing the risk of intraoperative cerebrospinal fluid leakage. Our study showed no significant difference in the incidence of intraoperative CSF leakage between the two groups, which may be related to the small sample size of our groups. Intraoperative CSF leakage does not necessarily mean postoperative CSF leakage. Rigorous skull base reconstruction can effectively prevent postoperative CSF leakage. Among the 41 patients in our study, 23 underwent intraoperative CSF leakage, but only 4 were diagnosed with postoperative CSF leakage.

ICA injury is considered the most dangerous intraoperative complication in this extended approach, although it has a low incidence (12, 15–17). No ICA injury was encountered in our cases, but once the ICA ruptures, the consequences are unimaginable. For intraoperative ICA injury, it is recommended to use vaseline gauze for emergency packing and compression hemostasis, and the assistant can press the injured ICA to push it to the ipsilateral cervical transverse process to reduce arterial blood flow and assist in hemostasis. Bipolar coagulation and muscle fascia packing can also be used for emergency treatment. DSA examination should be actively provided after the operation to prepare for subsequent treatment. Covered-stent placement is considered the best treatment for ICA injury at present, as it can close the blood vessels without blocking the blood flow (18, 19). We always believe prevention is more critical than the remedy for intraoperative ICA injury. Multiple intraoperative use of Doppler ultrasound to detect the ICA, following the correct surgical sequence, and carefully identifying and separating the relevant tissue structures that anchor the MW are the keys to preventing intraoperative ICA injury.

### 4.2 Microscopic dural invasion

In our study, the evidence of MW invasion was found in 71% (15/21) of the MW specimens, which is similar to the reports by Nagata et al. (69.2%) and Mohyeldin et al. (69%) and higher than the reports by Omar (43%) and Ishida (57%). This is speculated to be due to the inclusion of all patients with FPA in their studies, including those with no obvious evidence of MW invasion during surgery.

Interestingly, Nagata et al.'s study showed that one patient with Knosp grade 0 in their group found the presence of microscopic disease on histopathologic analysis, although with no evidence under the endoscope. Similar results were also obtained in Omar's study. These findings suggest that relying solely on the Knosp classification to judge tumor invasiveness may not be accurate. Studies by Dickerman et al. and Oldfield et al. suggest that the invasion of the parasellar dura, especially the medial wall of the cavernous sinus, may cause pituitary tumor recurrence.

It should be noted that the MW resection technique is primarily targeted at the invasion of the tumor into the MW. The result may not achieve the expectation when the tumor infiltrates other dura, excluding the MW, such as the lateral wall or the diaphragma sellae. In such cases, a combination of postoperative radiotherapy and chemotherapy may be required for comprehensive treatment. With the further understanding of skull base anatomy and the development of endoscopic technology, we believe that more surgical approaches and techniques can be explored to provide better solutions for invasive PAs.

### 4.3 Limitations of the study

This study aimed to introduce a technique of MW resection and evaluate the surgical effect. Limited by the sample size and follow-up time, the conclusions obtained may inevitably have selective bias, rendering our conclusions susceptible to random errors and extreme values. Additionally, a 12-month follow-up period may be insufficient to capture long-term outcomes, particularly for tumor recurrence. Furthermore, as this study is a single-center study and lacks external validation, the research findings may have limited generalization to other settings or populations, which restricts the scope of application of this study. A slight imbalance in sample size between the control and experimental groups may also contribute to the potential bias. To further validate the influence of this technique on biochemical remission and oncologic control, it is essential to conduct more extensive prospective studies in large randomized controlled trials with longer follow-up periods.

## 5 Conclusion

In this essay, we introduced a surgical technique for FPA with MW invasion. Applying this technique can effectively improve the biochemical remission rate and without increasing the surgical risk. Although the sample size in this study is relatively small and the follow-up time is relatively short, a large amount of data is still needed to confirm this technique further. However, we are convinced that the application of this surgical technique provides a new possibility for the treatment of FPAs.

## Data Availability

The original contributions presented in the study are included in the article/Supplementary Material, further inquiries can be directed to the corresponding author.
